# Possible Potentiation by Certain Antioxidants of the Anti-Inflammatory Effects of Diclofenac in Rats

**DOI:** 10.1155/2014/731462

**Published:** 2014-03-12

**Authors:** Samah S. Abbas, Mona F. Schaalan, Ashraf K. Bahgat, Ezzeddin S. El-Denshary

**Affiliations:** ^1^Department of Pharmacology and Toxicology, Misr International University (MIU), Km 28, Cairo-Ismailia Road, P.O. Box 1, Heliopolis, Cairo, Egypt; ^2^Department of Biochemistry, Faculty of Pharmacy, Misr International University (MIU), Km 28, Cairo-Ismailia Road, P.O. Box 1, Heliopolis, Cairo, Egypt; ^3^Department of Pharmacology and Toxicology, Faculty of Pharmacy, Cairo University, Giza, Egypt

## Abstract

In the present study, we investigated the potential beneficial impact of the addition of antioxidant supplements to diclofenac regimen in a model of carrageenan-induced paw. Rats were treated daily with antioxidants, that is, a-lipoic acid (50 mg/kg), selenium (2.5 mg/kg), vitamin C (1 g/kg), vitamin E (300 mg/kg), or zinc (25 mg/kg) on seven successive days and then received a single treatment with diclofenac or saline before carrageenan was injected to induce paw inflammation. The results indicated that these combinations did not significantly affect the percentage inhibition of paw edema caused by diclofenac alone; however, some combination treatments ameliorated signs of concomitant oxidative stress (such as alterations in plasma malondialdehyde (MDA) levels, hemolysate reduced glutathione levels, and erythrocytic superoxide dismutase enzyme activities) imparted by diclofenac alone. In some cases, few tested antioxidants in combination with diclofenac resulted in increased plasma levels of interleukin- (IL-) 6 and C-reactive protein (CRP). In conclusion, the results of these studies suggested to us that the added presence of natural antioxidants could be beneficial as standard anti-inflammatory therapeutics for a patient under diclofenac treatment, albeit that these effects do not appear to significantly build upon those that could be obtained from this common anti-inflammatory agent *per se*.

## 1. Introduction

During the last decade, great advances have been made for understanding the pathophysiology of inflammation and the involvement of reactive oxygen species (ROS) in its pathogenesis. Inflammation is a complex defense mechanism in which leukocytes migrate from the vasculature into damaged tissues to destroy the agents that can potentially cause tissue injury [1]. Millions of people all over the world are suffering from inflammatory disorders, making them use huge amounts of anti-inflammatory agents (i.e., diclofenac, a nonselective cyclooxygenase [COX] inhibitor) for many years in their lives. However, the serious side effects and the induced intolerance of the anti-inflammatory drugs have led to the search for methods in order to decrease their effective doses and improve their safety patterns.

A plethora of evidence has shown that overproduction of ROS occurs at sites of inflammation and this contributes to overall tissue damage. A subsequent oxidative stress (OS) predominates when production of ROS exceeds the capacity of cellular antioxidant defenses to remove these toxic species [2]. Due to their high reactivity, ROS are potentially causing damage to biomolecules such as DNA, lipids, and proteins. Thus, there is increasing interest in examining the potential benefits from providing patients antioxidants, such as *α*-lipoic acid (*α*-LA), selenium (Se), Vitamin C (Vit C), Vitamin E (Vit E), and zinc (Zn)-supplements, as “add-ons” to their diclofenac regimen.

Accordingly, the aim of this study was to investigate the ability of several vitamins and elements (commonly taken as food supplements) to reduce any deleterious side effects from diclofenac or to potentially bolster the desired effects from the drug. While our main interest was to screen the potential efficacy of these vitamins as stand-alone agents in place of diclofenac or as “add-ons” to diclofenac against an induced inflammation* in situ*, we also hoped to begin to define any potential mechanisms of action that these agents might utilize in bringing about the observed effects. This included analyses of effects of each agent alone/in combination with diclofenac on some key cytokines∖inflammatory mediators (i.e., interleukin [IL]-6 and C-reactive protein [CRP]) released during an inflammatory event). While we focused on the potential for these agents to reduce any undesired toxicities from the chronic use of diclofenac, we were also mindful that these studies could provide information that would allow for an unintended benefit potentially allowing for a decrease in the effective dose of diclofenac needed by a patient and a subsequent decrease in the risk of development of adverse effects.

## 2. Materials and Methods

### 2.1. Animals

Male Sprague Dawley rats (National Research Center, Giza, Egypt) weighing 100–120 g were used in this study. Rats were housed in a room with controlled temperature (18–22°C), humidity (60 ± 10%), and light/dark (12 hr/d) cycles for at least 1 week before being randomized into various experimental groups. Throughout the study, rats were provided* ad libitum *access to standard pellet chow (El-Nasr chemical Co., Cairo, Egypt) and filtered water. All experiments were conducted in accordance with the principles and procedures outlined in the International Guide for the Care and Use of Laboratory Animals and were approved by the Ethics Committee of the Faculty of Pharmacy Cairo University.

### 2.2. Drugs

Diclofenac (Diclofenac K; Novartis, Cairo, Egypt) was dissolved in distilled water to a concentration that would assure that a rat would receive* per os* a dose of 5 mg diclofenac/kg body weight. In these studies, the single diclofenac treatment occurred alone or in combination with (i.e., 2 hr after the final of the 7 daily doses) the test antioxidants. *α*-Lipoic acid (*α*-LA; EVA Pharma, Giza, Egypt; in normal saline) was delivered by intraperitoneal injection at a dose of 50 mg *α*-LA/kg [3]. Each daily* per os* delivery of sodium selenite (Sigma, St. Louis, MO; in water) at 2.5 mg sodium selenite/kg [4]; Vit C (ADWIC, Al Qalyubiyah, Egypt; in water) at 1 g/kg [5], Vit E (MP Biomedicals, Bas-Rhin, France; in olive oil) at 300 mg/kg [6], and zinc sulfate (ADWIC; in water) at 25 mg ZnSO4/kg [7] was performed without anesthesia and with a delivery volume of 1 mL/rat. The diclofenac volume (alone or after the final antioxidant dosing) was 1 mL/rat as well.

### 2.3. Experimental Design

Rats were randomly divided into 12 groups (*n* = 8/group): a carrageenan control, rats given diclofenac and then carrageenan, rats given Vit C, Zn, *α*-LA, Vit E, or Se daily for 7 d and then carrageenan, and rats given Vit C, Zn, *α*-LA, Vit E, or Se for 7 d, followed by diclofenac and then carrageenan. In the combination studies, treatment with diclofenac was always 2 hr after the final antioxidant/vehicle treatment; rats in the carrageenan-only and antioxidant-only groups received saline at this timepoint. The carrageenan injection was performed 2 hr later (i.e., 4 hr after the final daily administration with the test antioxidants (or vehicle)).

### 2.4. Induction of Inflammation

Rats were injected subcutaneously in the plantar side of their left hind paws with 0.05 mL of a 1% carrageenan solution. Footpad swelling was then assessed (by monitoring changes in pad thickness) using vernier calipers (Mitutoyo, Tokyo, Japan). Paw thickness was measured just before the injection and 1, 2, 3, and 4 hr after injection. Based on preliminary studies that showed that maximum paw thickness increase occurred at *≈*3 hr after injection of carrageenan; the values obtained at that timepoint were used here to calculate values of percentage inhibition of paw thickness increase.

### 2.5. Preparation of Blood Samples

Four hours after the carrageenan injection, 4 mL blood was collected from the retroorbital plexus into heparinized tubes. A 0.5 mL aliquot was immediately transferred to another heparinized tube for erythrocyte separation and for determination of superoxide dismutase (SOD) activity. A 100 *μ*L aliquot of the original blood was hemolyzed for determination of reduced glutathione (GSH) content. The remaining original blood was centrifuged (3000 rpm, 4°C, 15 min) to isolate plasma; this was stored at −20°C for subsequent determinations of levels of malondialdehyde (MDA), C-reactive protein (CRP), and IL-6.

### 2.6. Erythrocyte Separation

The aliquot spared for determination of SOD activity was centrifuged (3000 rpm, 4°C, 15 min); the precipitated RBC were washed with 3 mL cold saline and then centrifuged again at 3000 rpm for 10 min. After the supernatant was discarded, lysing of the RBC was performed by resuspension of the cells in 1.75 mL ice-cold distilled water and then vigorous shaking; the cells were then left to stand for 15 min at 4°C. The resulting hemolysate was used for determination of SOD activity.

### 2.7. Determination of Plasma Lipid Peroxides (MDA), CRP, and IL-6, Erythrocyte Superoxide Dismutase (SOD) Activity, and Blood Reduced Glutathione (GSH) Levels

The determination of lipid peroxide levels (as reflected in amounts of measurable MDA) was done as prescribed before [8]. In brief, to 0.2 mL plasma, 1.2 mL of 1% (w/v)* o*-phosphoric acid and 0.4 mL of 0.67% (w/v) thiobarbituric acid were added and mixed, and then the mixture was heated for 45 min in a boiling water bath. After cooling, 1.6 mL* n*-butanol was added and the sample was mixed vigorously. The butanol layer was separated by centrifugation at 3000 rpm for 15 min. The absorbance of the pink product in the butanol fraction was then measured at 535 and 520 nm in a Shimadzu double beam spectrophotometer (UV-150-02); all samples were read against a blank processed in parallel containing 0.2 mL distilled water instead of sample. The difference in absorbance between the two readings (i.e., Δ*A*_535–520_) was taken as a reflection of the level of MDA (nmol/mL) in the sample.

The pyrogallol autoxidation method was adopted for determination of erythrocyte SOD activity [9]. In brief, from the previously separated hemolysate of erythrocytes, 250 *μ*L was mixed vigorously with 0.75 mL chloroform-ethanol mixture (3 : 5 v/v) to precipitate hemoglobin in the sample. In a microcuvette, 1 mL Tris-HCl buffer (pH 8.2) was added to 30 *μ*L of 10 *μ*M pyrogallol solution and 100 *μ*L distilled water; the absorbance at 420 nm was then measured 30 and 90 sec thereafter. The difference in absorbance (Δ*A*) was used to reflect the rate of pyrogallol autoxidation (in 1 min) and was considered the experiment blank. The same procedure was then carried out using the prepared blood samples or standards containing SOD in the place of distilled water. The reduction or inhibition of rate of autoxidation in 1 min (compared to the blank) was used as an index of the SOD activity. The percentage change in pyrogallol autoxidation was calculated as follows: % change in pyrogallol autoxidation = 100 − 100 × (Δ*A*_*T*  or  *S*_/min⁡)/Δ*A*_*B*_/min⁡, where Δ*A*_*T*_/min⁡ = change in absorbance of the test sample in 1 min. ΔA_S_/min = change in absorbance of the standard sample in 1 min, and Δ*A*_*B*_/min⁡ = change in absorbance of the blank sample in 1 min.

Hemoglobin (Hb) was measured via the cyanomethemoglobin [10]. Hemoglobin in a sample aliquot (20 *μ*L) was converted to cyanomethemoglobin by addition of 5 mL Drabkin's reagent (0.6 mM potassium ferricyanide∖0.77 mM potassium cyanide); absorbance of the cyanomethemoglobin was then monitored at 540 nm. From these latter values, the concentration of Hb was calculated as follows: Hb concentration = *A*_sample_ × 36.77 (g/dL), where *A*_sample_ is sample absorbance at 540 nm, and 36.77 is a unitless constant factor. Based on these values, the SOD activities were then reexpressed as (U SOD/g Hb) in blood sample) according to the following equation: SOD activity in blood sample (U/g Hb) = SOD conc. (U/mL)/g Hb in sample.

GSH levels in blood were determined as prescribed before [11]. In brief, 1 mL of hemolysate (0.1 mL original blood + 0.9 mL distilled water) was combined with 1.5 mL of a protein precipitating solution (1.67 g* m*-phosphoric acid, 0.2 g EDTA, and 30 g NaCl in 100 mL distilled water) and then placed at room temperature for 5 min. The sample was then centrifuged (3000 rpm, 15 min) and the resultant supernatant assayed for GSH, that is, 1 mL supernatant (or standard GSH solution) was combined with 4 mL of the phosphate solution and 0.5 mL Ellman's reagent (40 mg 5,5′-dithiobis-(2-nitrobenzoic acid) [DTNB] in 100 mL 1% sodium citrate solution). The absorbance of the resulting yellow solution was then measured at 412 nm (within 5 min) in a spectrophotometer. From the absorbance values of standard GSH solutions, a standard curve was prepared and levels of GSH in each sample were then extrapolated (mg %).

Plasma CRP and IL-6 levels were measured according to the procedures described in IMMULITE CRP and IL-6 assay kits, respectively (Diagnostic Product Corporation (DPC), Los Angeles, CA). The sensitivity of the CRP and IL-6 assay kits—defined as the concentration two standard deviations above the response at zero dose—was *≈*1 pg/mL.

### 2.8. Statistical Analysis

Values were expressed as mean ± SD. Results were analyzed using one-way analysis of variance (ANOVA) followed by a least significant difference test (LSD) to compare between the different groups. A *P* value < 0.05 was accepted as significant in these tests. SPSS software (Chicago, IL) was used to carry out all analyses.

## 3. Results

### 3.1. Effects of Various Agents on the Rat Paw Edemas

Normal rats injected subcutaneously with* carrageenan* had an average 0.26 cm increase in paw thickness. Administration of diclofenac 2 hr prior to the carrageenan markedly inhibited induced edema by *≈*39%; that is, increase in paw thickness was 0.16 cm (Table 1). Daily administration of *α*-LA, Se, Vit C, Vit E, or Zn for 7 d prior to the injection of carrageenan significantly inhibited the induced paw edema by 23, 19, 19, 31, and 23%, respectively (i.e., thickness values were 0.20, 0.21, 0.21, 0.18, and 0.20 cm, resp.).

Administration of the following treatment combinations, diclofenac + *α*-LA, diclofenac + Se, diclofenac + Vit C, diclofenac + Vit E, or diclofenac + Zn, prior to injection of carrageenan markedly inhibited induced paw edema by 19, 27, 31, 27, and 46%, respectively, relative to those in hosts that had received no drug treatment (i.e., Group I). Increases in paw thicknesses in these hosts were 0.21, 0.19, 0.18, 0.19, and 0.14 cm, respectively (Table 2). The inhibitory effects imparted by the combinations were significantly no better than the diclofenac alone; only in the case of combination with Zn the inflammation was reduced better than the outcome induced by diclofenac alone.

### 3.2. Effects of Test Agents on Plasma MDA Levels

Injection of a 1% carrageenan solution into the paws of naive rats induced an oxidative stress reflected as a significant increase in plasma MDA; values in these rats were 2.77-fold above background (Tables 3 and 4). Rats that received diclofenac 2 hr before the carrageenan injection had a marked decrease in plasma MDA, that is, 56% relative to values in saline-treated carrageenan-injected rats. Daily injection of *α*-LA for 7 d prior to the carrageenan also caused a significant decrease in plasma MDA levels (47.6%) induced by the carrageenan itself. In rats that received a diclofenac + *α*-LA combination, the decrease in MDA levels (relative to carrageenan-only rat levels) reached 69.4%. Selenite administration for 7 d prior to induction of inflammation resulted in MDA levels being significantly lower (51.4%) than those in nondrug-treated counterparts; in comparison, the diclofenac + selenite combination significantly decreased MDA levels by just 40.7%. Treatment with Vit C for 7 d before induction of acute inflammation caused a significant 55.9% decrease in MDA levels; the diclofenac + Vit C combination significantly reduced (by 67.8%) MDA levels to values approximating those in normal rats. Vit E given for 7 d prior to inflammation induction also resulted in a significant 67.8% decrease in plasma MDA levels compared to the level in the carrageenan-only rats. Unlike with Vit C, the diclofenac + Vit E treatment was less effective than the antioxidant alone; that is, the change compared to the inflamed rat values was now only 40.0%. Lastly, dosing with Zn for 7 d before injection of carrageenan significantly decreased plasma levels of MDA (43.0%) relative to that in the nondrug-treated hosts; in this case, the additional presence of diclofenac resulted in a 69.5% reduction in MDA values.

### 3.3. Effects of Test Agents on Hemolysate GSH Levels and Erythrocyte SOD Activity

Significant decreases in hemolysate GSH levels and erythrocyte (RBC) SOD activity (72 and 86%, respectively, were noted in rats that received the carrageenan injection compared to values associated with the blood/cells of naïve [normal] rat) (Tables 3 and 4). Rats that received diclofenac 2 hr before injection of carrageenan had GSH levels and SOD activity that were significantly greater (5.70- and 2.90-fold, resp.) compared to those in the saline-injected inflamed rats. The daily *α*-LA regimen led to significant increases in the hemolysate GSH levels (2.15-fold versus control) and SOD activity (7.20-fold versus control) as well; the diclofenac + *α*-LA combination led to significant increases in hemolysate GSH and RBC SOD of 3.95- and 2.44-fold, respectively. Selenite treatments prior to induction of inflammation resulted in significant increases in the GSH (2.17-fold) and SOD activity (4.00-fold) levels; the diclofenac + selenite regimen led to a significant increase in hemolysate GSH (3.99 fold) levels, but the change in RBC SOD activity was insignificant as compared to the values for the carrageenan-only rats. Treatments with Vit C or Vit E for 7 d before induction of inflammation, each caused significant increases in hemolysate GSH (3.00- and 2.43-fold, resp.) and RBC SOD activity (4.75- and 2.60-fold, resp.) compared to values in the rats injected with carrageenan only. The additional presence of diclofenac resulted in significant increases in hemolysate GSH of *≈*4- and 2.45-fold for Vit C and Vit E, respectively; RBC SOD activity was increased by *≈*4- and 4.28-fold, respectively, as compared to levels in nondrug-treated inflamed rats. Lastly, Zn treatment prior to the carrageenan injection resulted in significant increases in hemolysate GSH levels (*≈*2-fold) and RBC SOD activity (4-fold) versus those in the carrageenan-only rats (Table 3). The diclofenac + Zn combination also led to significant increases in hemolysate GSH (*≈*3-fold) and RBC SOD activities (*≈*5-fold).

### 3.4. Effects of Test Agents on Plasma CRP and IL-6 Levels

**Figure 1:**
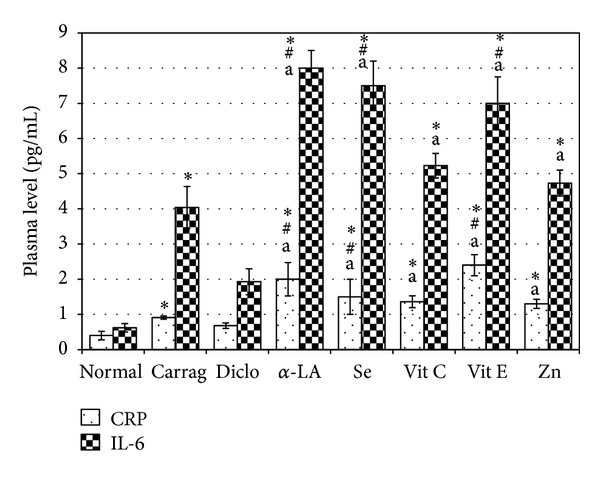
Effect of treatments on plasma CRP and IL-6 levels.All rats were provided daily oral doses of diclofenac (5 mg/kg), selenite (2.5 mg/kg), Vit C (1 g/kg), Vit E (300 mg/kg), or ZnSO_4 _(25 mg/kg); *α*-LA (50 mg/kg) was administered IP. ^∗#^Value is significantly different from normal control and carrageenan-only rats, respectively (*P* < 0.05). ^a^Value is significantly different from that of diclofenac-treated hosts (*P* < 0.05).

**Figure 2:**
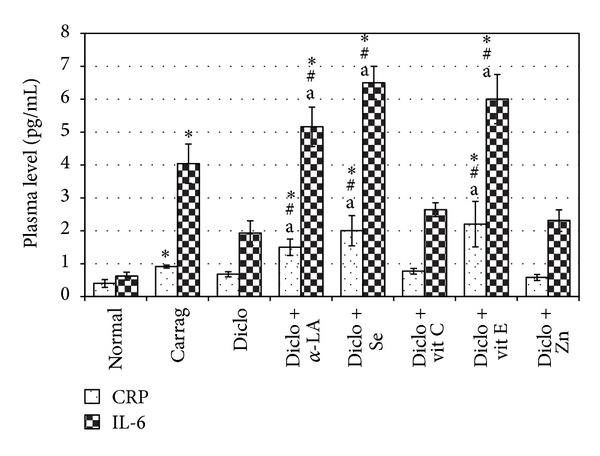
Effect of combinational treatments on plasma CRP and IL-6 levels. All rats were provided daily oral doses of diclofenac in combination with selenite, Vit C, Vit E, or ZnSO_4_; *α*-LA (50 mg/kg) was administered IP. Doses were the same as outlined in Figure 1 legend. ^∗#^Value is significantly different from normal control and carrageenan-only rats, respectively (*P* < 0.05). ^a^Value is significantly different from that of diclofenac-treated hosts (*P* < 0.05).

Levels of CRP and IL-6 in the plasma were also significantly increased (by 2.30- and 6.50-fold above naive level, resp.) as a result of carrageenan injection (Figures 1 [single agent treatments] and 2 [combination treatments]). Rats that received diclofenac 2 hr before injection of carrageenan had insignificant decreases in the “now-elevated” plasma levels of CRP and IL-6 (25.27 and 52.35% decrease, resp.) noted in the carrageenan-only-treated rats; interestingly, these latter levels were increased 2.30- and 6.50-fold, respectively, above naïve rat values. Daily *α*-LA injections prior to the carrageenan caused a significant increase in plasma levels of CRP (5.0- and 2.2-fold) and IL-6 (12.90- and 1.98-fold) compared to the levels in naive and carrageenan-only-treated rats, respectively (Figure 1). Rats that received the diclofenac + *α*-LAcombination also had significant increases in plasma CRP (3.77- and 1.65 fold) and IL-6 (8.30- and 1.28-fold) levels compared, respectively, to levels in naive and carrageenan-only rats (Figure 2). Selenite treatments also caused significant increases in plasma CRP (3.75- and 1.50-fold) and IL-6 (12.00- and 1.86-fold) levels; the diclofenac + selenite combination resulted in slightly greater significant increases in CRP (5.00- and 2.20-fold) and IL-6 (10.50- and 1.60-fold) levels. Vit C administration (alone or in combination with diclofenac) did not significantly affect plasma levels of CRP and IL-6 as compared to the levels in carrageenan-only rats. In contrast, Vit E given for 7 d prior to induction of inflammation resulted in significant increases in plasma CRP (6.00- and 2.60-fold) and IL-6 (12.00- and 1.86-fold) levels relative to levels in normal and carrageenan-only rats, respectively; the diclofenac + Vit E regimen caused slightly lower, albeit still significant, increases in CRP (5.5- and 2.4-fold) and IL-6 (9.7- and 1.5-fold) levels. Administration of Zn (alone or in combination with diclofenac) before the carrageenan did not significantly affect plasma CRP and IL-6 levels relative to those in the inflamed nondrug-treated rats.

## 4. Discussion

Interest in the relationship between inflammation and oxidative stress has risen in recent years as they share a common role in the etiology of a variety of chronic diseases. Many of these disorders share a common pathophysiological link in terms of chronic low-grade inflammation and overproduction of reactive oxygen and nitrogen species (ROS and RON). Based on this, studies were initiated to investigate the efficacy of several antioxidants to potentially reduce inflammation and cytokine mediators that occur in a model of acute inflammation (i.e., carrageenan-induced rat paw edema) [2].

In the study here, treating the acutely inflamed rats with diclofenac caused a significant inhibition in paw thickness increases, a finding previously noted using an oral dose dissimilar to that here [12]. Another study also showed that diclofenac was effective in reducing paw edema in both irradiated and nonirradiated rats using celecoxib [13]. The assessment here of the anti-inflammatory efficacy of antioxidants *α*-LA, Se, Vit C, Vit E, and Zn revealed a significant inhibition in paw thickness increases to different extents, that is, Vit E- > *α*-LA, Zn > Se∖Vit C. In all cases, these agents (alone or in combination with diclofenac) yielded changes in thickness increases significantly no better than the diclofenac alone; only in the case of combination with Zn the inflammation was reduced better than the outcome induced by diclofenac alone. This finding gave us a pause as to the potential significance of use of these antioxidants as* adjunctive* therapeutics. However, on their own, these agents did induce significant reductions in paw thickness; as such, our interest in their potential use as* stand-alone *anti-inflammatory agents remained intact.

Our findings with the antioxidants alone were in accordance with the study that investigated the anti-inflammatory effects of *α*-LA in carrageenan-paw edema model [3] and another study, which showed that administration of Vit C reduced expected increases in hind paw inflammatory edema in a model of adjuvant-induced arthritis in rats [5]. Abou-Mohamed et al. concluded that ZnSO4 supplementation reduced carrageenan-paw edema in rats as well [7].

An association between oxidative stress and inflammatory responses induced by carrageenan in our study was reflected in significant increases in plasma levels of MDA and decreases in both hemolysate GSH levels and RBC SOD activities. Such outcomes are similar to those reported by other studies [3, 14]. Another evidence indicated that diclofenac induced amelioration of elevated plasma MDA levels and increased the activity of total plasma SOD in a rat adjuvant arthritis model [15]. However, Khayyal et al. presented contrasting results; that is, diclofenac failed to alter plasma levels of MDA or affect GSH levels or SOD activity; it has been suggested that this contrasting outcome may be attributed to the small dose of diclofenac that these investigators used [13]. The antioxidant efficacy of the studied agents in terms of reduction of elevated MDA levels and elevation in both hemolysate GSH and erythrocyte SOD activity reiterates the findings of several previous studies [16–18]. Nevertheless, though the adjunctive therapy of the test agent with diclofenac failed to present any significant increased efficacy against changes in paw thickness edema measure, the findings still proved a significant promising in regard to antioxidant activity.

In general, plasma CRP levels correlate with severity of inflammatory disease or tissue injury [19]. In vitro studies have shown that control of this response is primarily regulated by IL-6 [20]. However, Jones et al. showed that the relationship between IL-6 and CRP is more complex than previously thought, since IL-6R shedding in response to CRP likely contributes to the formation of an agonistic sIL-6R/IL-6 complex [21]. Thus, CRP not only acts as an acute phase reactant but it also may have a profound effect on distal IL-6–mediated events that occur during the inflammatory process. Indeed, CRP levels in several diseases have been found to correlate with those of sIL-6R [22].

The consensus that the increase in plasma CRP and IL-6 levels correlates with severity of inflammatory disease was confirmed in the current study. Another study demonstrated an increase in IL-6 levels in blood 3 hr after injection of carrageenan in a model of carrageenan-induced hyperalgesia. Such outcomes are suggestive of a possibility that circulating IL-6 could act as a messenger of information from peripheral inflammatory sites to the CNS [23]. Furthermore, another study demonstrated that IL-6 serum levels were significantly increased at 24 hr following edema induction, but not after 3 hr, in a model of carrageenan-induced rat paw edema [24]. However, contradictory results indicated that LPS-induced inflammatory paw edema in rats, but not the type induced by carrageenan, resulted in measurable levels of IL-6 in serum within 3 hr of induction [25].

Oral administration of diclofenac here lowered the elevated plasma levels of CRP and IL-6, but the changes were not significant. Similarly, other investigators noted that circulating IL-6 levels remained unaffected after intra-arterial or peritoneal injection of diclofenac [26]. Moreover, investigating the modulatory effects of diclofenac on IL-6 and prostaglandin (PG) levels showed that diclofenac significantly decreased PGE2 production but had no significant effect on IL-6 levels [27]. As the anti-inflammatory effect of diclofenac was reflected only in the inhibition of the paw thickness increase as compared to that in the untreated inflamed rats but not in the decreases in plasma IL-6, it could be concluded that the cytokine inhibition does not completely explain the efficacy of cyclooxygenase (COX) inhibitors (like diclofenac) in downregulating acute inflammation (i.e., suggesting another mechanism independent on COX inhibition). This observation appears to confirm earlier observations [28, 29].

To our best understanding, the study here was the first attempt to test the additive anti-inflammatory impact on the studied test agents on diclofenac through assessment of CRP and IL-6. Interestingly, *α*-LA, Vit E, sodium selenite, and their combinations with diclofenac (contrary to with diclofenac monotherapy) caused significant increases in plasma levels of IL-6 and CRP as compared to levels measured in normal rats as well as in carrageenan-only-treated rats. Such immunostimulatory effects of these antioxidants, in particular, Vit E, have been demonstrated previously [30, 31].

A plethora of evidence indicates that CRP also performs anti-inflammatory functions* in situ *[32–37]. Moreover, it has been documented that IL-6 exhibits two contrasting features; it acts as a proinflammatory cytokine in models of chronic inflammatory diseases, that is, collagen-induced arthritis, murine colitis, or experimental autoimmune encephalomyelitis [38]. In contrast, in models of acute inflammation, IL-6 exhibits an anti-inflammatory profile [39]. It has also been reported that IL-6 is involved in T-cell activation and represents an essential competence factor that synergizes with IL-1 to control initial steps of T-cell activation, including induction of IL-2 and enhancement of responsiveness to IL-2 [40, 41]. As IL-2 production is dependent on the release of IL-6, and (of the agents tested here) Vit E supplementation increased IL-2 plasma levels [30, 31], this could explain the current interesting finding that Vit E caused a significant rise in plasma IL-6 levels in our inflamed hosts.

In conclusion, the results of the present study (when taken in the context of the above-noted studies) showed that the immunostimulatory effects of antioxidants, namely, *α*-LA, Vit E, and selenite, might be related to an induced release of IL-6 and subsequent induction of CRP release. The current results showed that the administration of other antioxidants, namely, Vit C, Zn, and their combinations with diclofenac, did not significantly affect plasma IL-6 and CRP levels. It is worth mentioning that the administration of diclofenac and *α*-LA, Se, and Vit E caused significant increases in plasma CRP and IL-6 levels as compared to values seen in untreated carrageenan-only injected rats. However, these levels were lower than those caused by administration of the each antioxidant individually.

In summary, the combination of diclofenac and any of the anti-inflammatory agents tested here appears to preserve any immunomodulating effect of the antioxidant alone. Thus, we conclude that the addition of antioxidants to any treatment regimen using this particular drug could have potential beneficial effects for the patient under treatment, albeit that it is not one that builds upon the effects from the diclofenac* per se*.

The presented findings are complementary to the conclusion drawn from the previously published systematic review of Canter et al. on evidenced randomized clinical trials (RCTs) for the effectiveness of the antioxidant Vitamins A, C, E, or selenium or their combination in the treatment of arthritis. Clinical trials testing the efficacy of Vitamin E in the treatment of arthritis have been methodologically weak and have produced contradictory findings. There is presently no convincing evidence that selenium, Vitamin A, Vitamin C, or the combination product selenium ACE are effective in the treatment of any type of arthritis [42].

## Figures and Tables

**Table 1:** Effects of diclofenac, *α*-LA, selenite, Vit C, Vit E, or zinc on paw thickness increase after 3 hr in a rat carrageenan-induced paw edema model.

Groups	Paw edema (thickness increase [cm] after 3 hr)	Inhibition %
Carrageenan (50 *μ*L of 1% solution)	0.26 ± 0.063	
Diclofenac (5 mg/kg, per os)	0.16 ± 0.001^#^	38%
*α*-LA (50 mg/kg, IP)	0.20 ± 0.047^#a ^	23%
Se (2.5 mg/kg, po)	0.21 ± 0.043^#a ^	19%
Vit C (1 g/kg, po)	0.21 ± 0.038^#a^	19%
Vit E (300 mg/kg, po)	0.18 ± 0.031^#a^	31%
ZnSO_4_ (25 mg/kg, po)	0.20 ± 0.044^#a^	23%

Values are means of 8 rats (±SD).

^#^Value significantly different from carrageenan control (*P* < 0.05).

^a^Value significantly different from diclofenac group (*P* < 0.05).

**Table 2:** Effects of combination treatments of diclofenac and the test antioxidants on paw thickness increase after 3 hr in a rat carrageenan-induced paw edema model.

Groups	Paw edema (thickness increase [cm] after 3 hr)	Inhibition %
Carrageenan (50 *μ*L of 1% solution)	0.26 ± 0.06	

Diclofenac (5 mg/kg, per os)	0.16 ± 0.001^#^	38%

Diclofenac + *α*-LA (50 mg/kg, IP)	0.21 ± 0.001^#a^	19%

Diclofenac + Se (2.5 mg/kg, po)	0.19 ± 0.03^#a^	27%

Diclofenac + Vit C (1 g/kg, per os)	0.18 ± 0.01^#a^	31%

Diclofenac + Vit E (300 mg/kg, po)	0.19 ± 0.04^#a^	27%

Diclofenac + Zn (25 mg/kg, po)	0.14 ± 0.001^#a^	46%

Values are means of 8 rats (±SD).

^#^Value is significantly different from carrageenan control (*P* < 0.05).

^a^Value is significantly different from diclofenac group (*P* < 0.05).

**Table 3:** Effects of diclofenac, *α*-LA, selenite, Vit C, Vit E, or zinc on plasma levels of MDA, hemolysate GSH, and erythrocyte SOD activity in a rat carrageenan-induced paw edema model.

Groups	MDA (nmol/mL)	GSH (mg %)	SOD (U/g Hb)
Normal (50 *μ*L saline)	0.21 ± 0.038	2.03 ± 0.21	3.95 ± 0.91
Carrageenan (50 *μ*L of 1% solution)	0.59 ± 0.06*	0.55 ± 0.26*	0.55 ± 0.21*
Diclofenac (5 mg/kg, per os)	0.26 ± 0.05^#^	3.16 ± 0.34^∗#^	1.61 ± 0.16^∗#^
α-LA (50 mg/kg, IP)	0.31 ± 0.09^∗#^	1.19 ± 0.26^∗#a^	3.93 ± 0.87^#a^
Se (2.5 mg/kg, po)	0.29 ± 0.02^∗#^	1.59 ± 0.12^∗#a^	2.10 ± 0.44^∗#a^
Vit C (1 g/kg, po)	0.26 ± 0.03^#^	1.69 ± 0.30^∗#a^	2.59 ± 0.32^∗#a^
Vit E (300 mg/kg, po)	0.19 ± 0.03^#a^	1.35 ± 0.07^∗#a^	1.44 ± 0.25^∗#^
ZnSO_4_ (25 mg/kg, po)	0.33 ± 0.05^∗#a^	1.20 ± 0.16^∗#a^	2.23 ± 0.57^∗#a^

Values are means of 8 rats (±SD).

*Value is significantly different from normal control (*P* < 0.05).

^#^Value is significantly different from carrageenan control (*P* < 0.05).

^a^Value is significantly different from diclofenac group (*P* < 0.05).

**Table 4:** Effects of combination treatments of diclofenac and the test antioxidants on plasma levels of MDA, hemolysate GSH, and erythrocyte SOD activity in a rat carrageenan-induced paw edema model.

Groups	MDA (nmol/mL)	GSH (mg %)	SOD (U/g Hb)
Normal (0.05 mL, 1% saline)	0.21 ± 0.05	2.03 ± 0.21	3.95 ± 0.91
Carrageenan (50 *μ*L of 1% solution)	0.59 ± 0.06*	0.55 ± 0.26*	0.55 ± 0.21*
Diclofenac (5 mg/kg, po)	0.26 ± 0.048^#^	3.16 ± 0.34^∗#^	1.61 ± 0.16^∗#^
Diclofenac + *α*-LA (50 mg/kg, IP)	0.18 ± 0.03^#a^	2.19 ± 0.18^#a^	1.33 ± 0.27^∗#^
Diclofenac + Se (2.5 mg/kg, po)	0.35 ± 0.08^∗#a^	2.21 ± 0.17^∗#a^	0.51 ± 0.28^∗a^
Diclofenac + Vit C (1 g/kg, po)	0.19 ± 0.02^#a^	2.20 ± 0.30^#a^	2.12 ± 0.55^∗#a^
Diclofenac + Vit E (300 mg/kg, po)	0.36 ± 0.09^∗#a^	1.36 ± 0.22^∗#a^	2.33 ± 0.39^∗#a^
Diclofenac + Zn (25 mg/kg, po)	0.18 ± 0.03^#a^	1.62 ± 0.20^∗#a^	2.65 ± 0.21^∗#a^

Values are means of 8 rats (±SD).

*Value is significantly different from normal control (*P* < 0.05).

^#^Value is significantly different from carrageenan control (*P* < 0.05).

^a^Value is significantly different from diclofenac group (*P* < 0.05).
